# How Contemporary Human Reproductive Behaviors Influence the Role of Fertility-Related Genes: The Example of the P53 Gene

**DOI:** 10.1371/journal.pone.0035431

**Published:** 2012-04-20

**Authors:** Rosa Maria Corbo, Giuseppe Gambina, Renato Scacchi

**Affiliations:** 1 Department of Biology and Biotechnology “Charles Darwin,” La Sapienza University, Rome, Italy; 2 Alzheimer's Disease and Other Dementias Center, Division of Neurology, Hospital of Verona, Verona, Italy; 3 CNR Institute of Molecular Biology and Pathology, Rome, Italy; National Cancer Institute, United States of America

## Abstract

Studies on human fertility genes have identified numerous risk/protective alleles involved in the occurrence of reproductive system diseases causing infertility or subfertility. Investigations we carried out in populations at natural fertility seem to suggest that the clinical relevance that some fertility genes are now acquiring depends on their interaction with contemporary reproductive behaviors (birth control, delayed childbearing, and spacing birth order, among others). In recent years, a new physiological role in human fertility regulation has emerged for the tumor- suppressor p53 gene (P53), and the P53 Arg72Pro polymorphism has been associated with recurrent implantation failure in humans. To lend support to our previous observations, we examined the impact of Arg72Pro polymorphism on fertility in two samples of Italian women not selected for impaired fertility but collected from populations with different (premodern and modern) reproductive behaviors. Among the women at near-natural fertility (n = 98), the P53 genotypes were not associated with different reproductive efficiency, whereas among those with modern reproductive behaviors (n = 68), the P53 genotypes were associated with different mean numbers of children [Pro/Pro = 0.75<Pro/Arg = 1.7<Arg/Arg = 2, (p = 0.056)] and a significant negative relationship between the number of children and P53 Pro allele frequencies (p = 0.028) was observed. These results are consistent with those of clinical studies reporting an association between the P53 Pro allele and recurrent implantation failure. By combining these findings with previous ones, we suggest here that some common variants of fertility genes may have become “detrimental” following exposure to modern reproductive patterns and might therefore be associated with reduced reproductive success. Set within an evolutionary framework, this change could lead to the selection of a set of gene variants fitter to current reproductive behaviors as the shift to later child-bearing age in developed countries.

## Introduction

Human fertility is a complex trait determined by gene-environmental interactions in which genetic factors represent a significant component [1], [2]. Fertility is regulated by a large number of genes acting in various different steps to establish both male and female fertility [3], [4]. Besides the numerous rare mutations associated with infertility, many polymorphic variants (alleles) have also been identified in fertility genes, but how these alleles affect human fertility remains unclear [3], [4].

Different alleles at a locus are usually thought to be associated only with small fertility differences (or no differences at all) and contribute very little to overall fitness, otherwise selection would lead to their fixation or elimination [5], [6]. This scenario would fit well with the complex trait model proposed for fertility which predicts the contribution of many genes, each with a small effect. In recent investigations we were able to examine the way the variation of some fertility-related genes may influence reproductive efficiency in natural (or near-natural) fertility populations (i.e. in the absence of deliberate birth control) [7], [8] and could confirm the absence of a relevant effect of gene variants on total fertility (i.e., fertility of subjects at the end of their reproductive lifetime). Other recent studies on fertility candidate genes have identified risk/protective alleles involved in the occurrence of reproductive system diseases causing infertility or subfertility [9]–[12]. More recently, some of these polymorphisms have been studied also in relation to their role in positive outcomes after *in vitro* fertilization (IVF) or other assisted reproductive technology (ART) procedures [13]–[15]. The emerging picture seems to indicate that fertility genes represent a set of genes whose role is changing, and acquiring clinical relevance, a phenomenon we hypothesize may be due to their interaction with the present environmental context, i.e., modern reproductive patterns (e.g., birth control, family planning, delayed childbearing, and spacing birth order) followed in many of today's industrialized countries.

Whether for economic, social, or educational reasons, young couples have begun to postpone starting a family and reduce family size, and women to delay having first childbirth or decide to remain childless. In Western Europe, the mean age at which women deliver their first child rose from 22–25 years in 1950 to 27–30 years in 2003 [16], i.e., later in life when fertility starts to decline. Studies on age-related female fertility indicate that it begins to decline at age 30 years and then more rapidly after age 35. While the decline in male fertility is also age-dependent, it becomes significant only after age 45 years [17]. Furthermore, postponement of first childbirth is accompanied by increased serious health risks for mothers and their children. A major effect of this shift in reproductive behaviors is a progressive reduction of fertility. Across Europe, total fertility rates ([TFR], average number of children per woman) decreased dramatically from 2·6 between 1960 and 1964 to 1·5 between 2004 and 2005, a value well below 2·1, the TFR needed for generation replacement, i.e. the level of fertility at which a population exactly replaces itself from one generation to the next (http://ec.europa.eu/employment_social/social_situation/docs/sec_2007_638_en.pdf; last accessed February 23, 2012).

To verify the hypothesis that exposure to different reproductive behaviors may influence the action of fertility-related genes, we examined the role of another fertility gene, P53, in two samples of the Italian population with different reproductive patterns. The tumor-suppressor p53 gene (P53) has a pivotal role in tumor prevention and DNA damage response. In response to a variety of cellular stresses, the p53 protein (p53) is able to activate target genes to induce cell cycle arrest, apoptosis, and senescence. Through these mechanisms, p53 promotes DNA repair or the elimination of damaged cells, thus contributing to tumor suppression [18].

In the last years, a new physiological role has emerged for p53 as a fertility regulator [19]. In mice maternal reproduction p53 was found to control the implantation of fertilized eggs, a function p53 exerts by activating leukemia inhibitory factor (LIF), an essential gene for blastocyst implantation [20]. So too in humans LIF appears to be involved in egg implantation, given that LIF levels are significantly decreased in women with unexplained infertility [21]. The role of p53 in the regulation of female fertility has been confirmed by more recent observations of how other members of the p53 family (p63 and p73) participate in the control of human maternal reproduction [22].

Many single nucleotide polymorphisms (SNPs) of P53 have been identified in human populations, but the one most extensively studied is rs1042522, located in codon 72 of exon 4, where the change CGC→CCC results in an Arg/Pro substitution. The Pro and Arg alleles at codon 72 are not functionally equivalent: Pro72 encodes a weaker p53 isoform than that encoded by Arg72 to induce apoptosis and suppress transformation, while it is better at favoring cell cycle arrest [23]. The two alleles at P53 codon 72 differ also in their transcriptional activity toward a subset of p53-responsive genes. Notably, cells containing P53 Arg72 produce twice as much LIF as those containing the Pro72 allele [24]. A growing body of epidemiological data is consistent with these *in vitro* studies: the P53 Pro72 allele was found at increased frequencies among women undergoing *in vitro* fertilization (IVF) and, among such women, it was associated with higher implantation failure [24], [25]. This clinical observation confirms that p53 regulates maternal reproduction in humans as well.

So far, the P53 Arg72Pro polymorphism has been found to influence human reproduction in a pathological context. We examined it in two samples of women not selected for impaired fertility and collected from populations with different reproductive patterns. The first was collected from a rural population from Southern Italy, which, according to population and sample fertility rate, has reproduced with a pre-modern or near-natural reproductive pattern. The second was collected from an urban population from northeastern Italy whose fertility rate indicates a pattern of reproduction typical of modern societies where birth control is practiced.

The aim of the present study was to examine the role of P53 polymorphism in fertility at the population (not clinical) level and the possible effects different reproductive behaviors may have on it.

## Results

The characteristics of the samples are reported in Table 1. The observed fertility rate for the women in the Southern Italian sample was 3.7±2.3 children (range, 0–11). This value was intermediate with respect to the range of 4.8-3.1 reported for cohorts of women born in the same geographic area and in the same period (1900–1930) [26]. The fertility rate for the women in the Northern Italian sample was 1.8±0.97 children (range, 0–4). This value was near the lower end of the range of 2.4–1.7 reported for cohorts of women born in the same geographic area and in the same period (1921–1953) [27]. Spontaneous abortion rates were calculated as the ratio between the number of abortions and the number of children. No population data concerning cohorts of women of the same periods are available for a comparison with the sample data; however, the abortion rates (abortion/livebirths) reported for the same geographic areas (Northern Italy, 0.13) and (Southern Italy, 0.12) are available for the period 1997–2001 [http://www3.istat.it/dati/catalogo/asi2004/PDF/Cap3.pdf, last accessed February/27/2012].

**Table 1:** Characteristics of the two study samples.

	Southern Italy	Northern Italy
**Age (yrs)** *	82.2±6.0	70.3±9.4
**Total no. of children**	342	119
**Mean no. of children** *	3.7±2.3	1.8±0.97
**Total no. of abortions**	65	6
**Abortion/children ratio**	0.19	0.05
**Childless subjects (%)**	7.7	11.8

* Plus-minus value is equal to plus-minus standard deviation.

The P53 Arg72Pro polymorphism was examined in the Southern Italian (n = 152) and the Northern Italian (n = 102) samples. The observed genotype frequencies (Southern Italy: Pro/Pro 0.112, Pro/Arg 0.382, Arg/Arg 0.507; Northern Italy: Pro/Pro 0.069, Pro/Arg 0.363, Arg/Arg 0.568) agreed with those expected according to the Hardy-Weinberg equilibrium. The P53 Pro allele frequencies of the two samples were 0.250 (Northern Italy) and 0.303 (Southern Italy), similar to those reported for the Italian population [28]. Table 2 reports the mean number of children associated with P53 genotypes in the two samples. Among the women in the Southern Italian sample, the P53 genotypes were not found to be associated with a different number of children. In contrast, among the women in the Northern Italian sample, the P53 genotypes were associated with different numbers of children, with a trend among the genotypes (from the lowest number of children to the highest) Pro/Pro<Pro/Arg<Arg/Arg (p = 0.056). The power calculation of this analysis is low (1-β<0.80) and the probability to make a type II error is high (β>0.20). We calculated that we would need to have approximately 50 subjects in each class to reach a statistical power of 0.80. But because of the P53 Pro allele frequency, we would have to collect about 800 subjects to find about 50 Pro/Pro homozygotes.

**Table 2:** Mean number of children (±SD) according to P53 genotype. The number of subjects is given in brackets.

P53 genotype	Southern Italy	Northern Italy
**Pro/Pro**	3.6±2.2(11)	0.75±1.0 (4)
**Pro/Arg**	3.6±2.1 (30)	1.67±0.9 (21)
**Arg/Arg**	3.7±2.6 (50)	1.95±1.0 (38)
**p**	0.98	0.056

A statistically significant result was achieved with a different approach: the subjects were grouped into different classes according to the number of children, and the P53 allele frequencies were calculated for each class (Table 3). The chi square test for trend of proportions showed a significant negative relationship between the number of children and P53 Pro allele frequencies (chi square = 4.9,d.f. = 1, p = 0.028) only in the Northern Italian sample, where the highest Pro allele frequencies were found in the class with no children (0.438) and the lowest in the class with two or more children. No significant trend was observed among the women in the Southern Italian sample (chi square = 0.11,d.f. = 1, p = 0.74). In neither sample were differences observed in the mean number of spontaneous abortions associated with the P53 genotypes (Southern Italian sample, p = 0.40; Northern Italian sample, p = 0.62) (data not reported).

**Table 3:** P53 allele frequencies in subjects grouped according to number of children.

No. of children	Northern Italy		Southern Italy	
	No. of subjects	Pro freq.	No. of subjects	Pro freq.
**0**	8	0.438	7	0.214
**1**	13	0.231	13	0.231
**2**	29	0.224	9	0.389
**3**	11	0.136	17	0.324
**4**	2	0.0	12	0.250
**>4**	0	0.0	33	0.287
**p**	0.027		0.74	

## Discussion

This study examined the association of the P53 Arg72Pro polymorphism with fertility at the population level, specifically in women not selected for fertility disorders. The data show that in the sample of women at near-natural fertility, the P53 genotypes are not associated with differential fertility. Conversely, in the sample of women with modern reproductive behaviors, the P53 genotypes are associated with a different reproductive efficiency, with the lowest fertility occurring among the Pro/Pro genotype carriers. The observation of a reduced fertility associated with the Pro allele was confirmed by subsequent chi square for trend of proportions test that shows a significant inverse relationship between Pro allele frequency and classes according to number of children.

These results should be interpreted with caution. The size of the samples is relatively small and, fertility being a complex trait, numerous other genetic and environmental factors, including male fertility, could have contributed to determine the phenotype “number of children” we examined. Nevertheless, the results obtained in the sample of women with a modern reproductive pattern are consistent with those of clinical studies reporting an excess of P53 Pro/Pro genotypes in women with unexplained infertility undergoing IVF and, among these, in women with higher implantation failure [24], [25]. The observation that P53 genotypes are not associated with different numbers of spontaneous abortions, which, by definition, represent the end of an established pregnancy, is not unexpected, given that P53 is involved in egg implantation instead.

It is interesting to observe how the phenotypic effects of P53 genotypes vary according to reproductive environments. In women from the population at near-natural fertility, P53 genotypes do not influence fertility, suggesting that the possible small reduction of fertility associated with the P53 Pro allele could have been offset by the pre-modern reproductive behavior (little or no birth control, lower age at first birth, etc.). In the sample from the population where modern reproductive behaviors began to be followed, the P53 genotype effect begins to shift toward differential fertility. Set within the broader context of contemporary society, the role of P53 genotypes eventually tends to acquire clinical relevance, being associated with infertility and the successful/or not outcome in women undergoing IVF [24], [25].

These findings are similar to what we have observed for other fertility-related genes. One gene we studied was ESR1, which encodes estrogen receptor alpha [7]. We examined the association of two ESR1 polymorphisms (rs 2234693, rs9340799) with fertility in two populations at near-natural (Italian) and natural (Afro-Ecuadorian) fertility and observed that the effects of ESR1 genotypes on fertility varied according to the reproductive environment: in the women from the African-Ecuadorian populations ESR1 genotypes (*pp* and *xx*) influenced the fertility level, predisposing to have an elevated number of children (higher than the median value). In the Italian women, the same genotypes showed an association with a low rate of spontaneous abortions, but without any impact on total fertility. In today's industrialized societies, however, the effects of the ESR1 genotype manifest as a successful outcome in women undergoing IVF [13], [14] or predisposing to premature ovarian failure [12], or exerting a protective effect against azoospermia or idiopathic oligospermia in men [11]. In other words, in populations with pre-modern reproductive patterns, the ESR1 genotypes influence at most the number of children, whereas within the context of modern reproductive behaviors, they seem to be associated instead with having children or not.

Another example is the angiotensin I-converting enzyme (ACE) gene. Recently, the ACE I/D polymorphism was found associated with recurrent spontaneous miscarriages [29]–[31]. We analyzed the relationship between the ACE I/D polymorphism and the number of spontaneous abortions, the number of pregnancies and completed fertility in a sample of Italian women at near-natural fertility [8]. We were able to tease out in this population sample an association between an ACE genotype (DD) and recurrent miscarriages, as previously reported in case-control studies [29]–[31]; however, no difference in completed fertility was observed among the women carrying the different ACE genotypes, as those carrying the ACE risk genotype and prone to miscarriage overcame the problem by counterbalancing it with a greater number of pregnancies. In contrast, published data on contemporary women appear to indicate that the ACE genotype could now be associated with reduced reproductive success, presumably because of their shorter reproductive lifespan due to postponement of childbearing. Similarly, other fertility related genes which do not seem to have any effects on fertility in populations with pre-modern reproductive patterns have been recently associated with infertility or ovarian stimulation outcome [32]–[33] (Table 4).

**Table 4:** Fertility related polymorphic genes which have been investigated for their role in populations with different reproductive patterns.

Gene	Phenotypes		Ref
	Premodern pattern	Modern pattern	
**ESR1**	fertility level	IVF outcome; POF^a^	7, 12–15
**FSHR**	none	IVF outcome	15,32, unpubl.data.
**CYP19**	none	IVF/COH^a^; POF^a^	15,33
**P53**	none	infertility; IVF outcome	Present invest.,24, 25
**ACE**	none (see text)	recurrent miscarriages.	8, 29–31

a POF:premature ovarian failure; COH: controlled ovarian hyperstimulation.

The emerging picture of the interactions between fertility genes and contemporary reproductive behaviors suggests that fertility genes may represent a further example of genes “rendered detrimental by progress”. The hypothesis for a “genotype rendered detrimental by progress” was first advanced by J.V. Neel [34] in 1962 in an attempt to explain the emergence of diabetes epidemics in the United States and other technologically advanced societies. He speculated the existence of a “thrifty genotype”, i.e., a set of genes shaped for our hunter-gatherer ancestors to survive on an intermittent and unpredictable food supply and with elevated physical activity. Such a genotype would be exceptionally efficient in the utilization of food and allow the storage of fat reserves in times of plenty for later times of shortage. Once highly advantageous for ensuring survival, these thrifty genes have become detrimental in modern societies where food is plentiful or overabundant and overall physical activity has declined. Moreover, they may be identified with susceptibility genes for complex diseases like type 2 diabetes or obesity. The notion that an “ancient” genotype could increase disease susceptibility has been put forth to account for the growing prevalence of such complex disorders as hypertension, coronary heart disease and Alzheimer's disease. Some 30 years later, in 1998 Neel suggested broadening the original concept to include the “civilization syndromes” or the “altered lifestyle syndromes” for diseases which can be explained by a similar evolutionary framework [35]. This hypothesis, originally based on disease physiology and epidemiology, found recent support from population genetic studies showing that, for several susceptibility polymorphisms for common disease, the risk allele is the ancestral allele, the result of an ancient adaptation no longer advantageous in the modern environment [36].

Taken together, our data seem to indicate that fertility genes might easily fit within the “altered lifestyle syndromes” hypothesis, and, at least for the P53 and ACE genes, in the ancestral susceptibility model as well, given that both the Pro allele of P53 and the D allele of ACE are the ancestral alleles [36], [37]. There is, however, an important difference between fertility genes and other genes “rendered detrimental by progress”: the latter are mostly susceptibility genes for diseases occurring in post-reproductive age (coronary heart disease, hypertension, Alzheimer's disease, among others) and therefore escape selection, while the natural fertility gene variants may be associated with differential fitness and, hence, be under selective pressure. This process could promote the selection of a set of gene variants fitter to the current reproductive patterns, thus favoring, for example, reproduction at a later age as well.

On the whole, the picture we have outlined suggests the occurrence of complex interactions between genetic factors and reproductive environments and that fertility genes are a further set of genes whose functions are influenced by the lifestyle changes of modern societies.

## Materials and Methods

### Study populations

The Southern Italian population sample included 152 apparently healthy unrelated women in the post-reproductive age collected for a population survey of the elderly population of Cilento, a rural area in the district of Salerno, Southern Italy. All subjects were born before 1930 (1900–1930) and lived in a society where the demographic transition (increase in life expectancy and declining fertility) had just started [38]. In Italian women born in the same geographic area and in the same year range, the mean age at marriage was 22.4 years [26], and the fertility rate was well over the replacement level (range, 4.8-3.1). All subjects were recruited consecutively without selection criteria, except age (>70 years) and birthplace. Fertility data (number of children) were ascertained by interviews and were collected for only 98 women. Written informed consent was obtained from all subjects. The protocol for the collection of biological material for the scientific studies was approved by the institutional committees (Local Health Unit ASL SA 3).

The Northern Italian population sample comprised 102 healthy unrelated women recruited as a control sample during an epidemiological study on Alzheimer's disease (AD) carried out at the Verona Hospital. All subjects were born between 1921/1953 in the urban area of Verona, northeastern Italy, and lived in a society where the demographic transition was already under way. In the Italian women born in the same geographic area in the same year range, the mean age at marriage was 25.2 years and the fertility rate was near/below the replacement level (range, 2.4-1.7) [27]. All subjects were recruited consecutively without selection criteria, except age that matched that of AD patients (>60 years) and birthplace (Verona province). Fertility data (number of children) were ascertained by interviews and were collected for only 63 women. Written informed consent was obtained from all subjects. The protocol for the collection of biological material for the scientific studies was approved by the institutional ethics committee (Hospital of Verona, Ethical Committee protocol no. 1268/131/2006). The study was approved by the Department Board (12/06/2009 session) of the former “Department of Genetics and Molecular Biology” of La Sapienza University, Rome.

### Laboratory Methods

Venous blood was drawn in ethylenediaminetetraacetic acid (EDTA) from all subjects after overnight fasting. High-molecular-weight DNA was extracted from whole blood according to the procedure described by Miller et al. [39]. The P53 SNP (rs1042522) was investigated by an allele-specific PCR according to Giannoudis et al. [40]. In brief, P53 Arg and Pro sequences were amplified in a single reaction with an allele-specific primer pair: P53 Arg sequences were detected using the primer pair p531, 58-GTCCCCCTTGCCGTCCCA-38, and Arg–, 58-CTGGTGCAGGGGCCACGC-38, amplifying a 137-bp fragment; P53 Pro sequences were detected using the primer pair Pro1, 58-GCCAGAGGCTGCTCCCCC-38 and p53–,8-GGAAGCCAGCCCCTCAGG-38, amplifying a 206-bp fragment. Visualization of the PCR product on agarose gel revealed three different genotypic patterns.

### Statistical Analysis

Allelic frequencies were determined by the gene-counting method. The agreement of the genotype distribution with Hardy–Weinberg expectations was verified with the chi square test. Comparisons between the mean number of children associated with different P53 genotypes were performed using analysis of variance (ANOVA). Estimates of statistical power (1-β) were calculated according to Cohen [41] i.e. we calculated the probability of rejecting the null hypothesis, when the null is false. In this analysis the null hypothesis is that the P53 genotypes are associated with the same number of children. The Cochran-Armitage chi square test for trend of proportions [42] was used to verify the presence of a linear trend between the number of children and P53 allele frequencies.
